# Supporting shared decision making for older people with multiple health and social care needs: a realist synthesis

**DOI:** 10.1186/s12877-018-0853-9

**Published:** 2018-07-18

**Authors:** Frances Bunn, Claire Goodman, Bridget Russell, Patricia Wilson, Jill Manthorpe, Greta Rait, Isabel Hodkinson, Marie-Anne Durand

**Affiliations:** 1 Centre for Research in Public Health and Community Care, University of Hertfordshire, College Lane, Hatfield, Hertfordshire, AL10 9AB UK; 2 Centre for Health Service Studies, University of Kent, George Allen Wing, Canterbury, Kent CT2 7NF UK; 3 Social Care Workforce Research Unit, King’s College London, Strand, London, WC2B 4LL UK; 4 Research Department of Primary Care and Population Health, UCL Medical School (Royal Free Campus), Rowland Hill Street, London, NW3 2PF UK; 5 Tower Hamlets Clinical Commissioning Group, The Tredegar Practice, London, E3 5JD UK; 6 The Preference Laboratory, The Dartmouth Institute for Health Policy & Clinical Practice, Level 5, Williamson Translational Research Building, Lebanon, New Hampshire USA

**Keywords:** Shared decision making, Person-centred care, Realist synthesis, Multimorbidity, Older people

## Abstract

**Background:**

Health care systems are increasingly moving towards more integrated approaches. Shared decision making (SDM) is central to these models but may be complicated by the need to negotiate and communicate decisions between multiple providers, as well as patients and their family carers; particularly for older people with complex needs. The aim of this review was to provide a context relevant understanding of how interventions to facilitate SDM might work for older people with multiple health and care needs, and how they might be applied in integrated care models.

**Methods:**

Iterative, stakeholder driven, realist synthesis following RAMESES publication standards. It involved: 1) scoping literature and stakeholder interviews *(n* = 13) to develop initial programme theory/ies, 2) systematic searches for evidence to test and develop the theories, and 3) validation of programme theory/ies with stakeholders (*n* = 11). We searched PubMed, The Cochrane Library, Scopus, Google, Google Scholar, and undertook lateral searches. All types of evidence were included.

**Results:**

We included 88 papers; 29 focused on older people or people with complex needs. We identified four context-mechanism-outcome configurations that together provide an account of what needs to be in place for SDM to work for older people with complex needs. This includes: understanding and assessing patient and carer values and capacity to access and use care, organising systems to support and prioritise SDM, supporting and preparing patients and family carers to engage in SDM and a person-centred culture of which SDM is a part. Programmes likely to be successful in promoting SDM are those that allow older people to feel that they are respected and understood, and that engender confidence to engage in SDM.

**Conclusions:**

To embed SDM in practice requires a radical shift from a biomedical focus to a more person-centred ethos. Service providers will need support to change their professional behaviour and to better organise and deliver services. Face to face interactions, permission and space to discuss options, and continuity of patient-professional relationships are key in supporting older people with complex needs to engage in SDM. Future research needs to focus on inter-professional approaches to SDM and how families and carers are involved.

**Electronic supplementary material:**

The online version of this article (10.1186/s12877-018-0853-9) contains supplementary material, which is available to authorized users.

## Background

Shared decision making (SDM) involves patients and health and social care practitioners (HSCPs) jointly offering treatment, care and support packages to reflect, respect and accommodate the patient’s preferences, priorities and goals [1, 2]. Although the original underlying ethos for sharing decisions between patients and HSCPs is based on values, i.e. people have the right to self-determination and autonomy, there is evidence that SDM can lead to better outcomes and care for people [3]. For example, patients who feel involved in the decision and in accord with the HSCP are less likely to need other services such as extra tests or referrals to other HSCPs [4]. More recently SDM has been envisaged as part of person and family centred care and integrated care [5–16].

Decision making becomes more complex for older people with multiple health and care needs as the capacity to self-manage is affected by the cumulative effects of long-term conditions. The nature of decisions is complicated by resource availability, polypharmacy, decline in decision making abilities and concordance, availability of support networks, suitability of treatment, safeguarding and the increased likelihood of depression [17–20]. Moreover, decision making may need to be negotiated between, and communicated to, multiple health and social care practitioners, as well as patients and their families. Whilst there is evidence that many older people and their family carers would like to be involved in decision making [21, 22], there is little evidence relating specifically to SDM with older people with complex health needs.

The skills for sharing and discussing personal information with vulnerable patients, and their families, can be hard to embed in services. There is a need to establish the mechanisms that preserve and foster shared decision making (SDM) between professionals, patients and carers and how they achieve improvements in patient outcomes [23, 24]. Approaches are needed that aim to address the complexity of life when living with, and managing, multiple long-term conditions [25, 26] or recognise the need to consider the abilities of patients and their families to attend to the demands of each condition [19, 27, 28]. Such approaches require the building of relationships, meaningful discussion and SDM between a range of different providers, patients and carers [29].

To develop an understanding of the realities of working in and across complex, overlapping systems of care, it is necessary to synthesise evidence from diverse strands of research [30, 31]. Realist methodology allows the deconstruction of component theories underpinning different interventions and enables us to consider relevant contextual data to test our understanding of the applicability of different approaches for older people with multi-morbidity and how SDM might achieve desired outcomes such as improvements in; patient safety, clinical effectiveness, quality of life and patient experience [23] within the context of integration. The aim of this review was to develop an explanatory account of how interventions to facilitate SDM might work for older people with multiple health and care needs, and how they might be applied to integrated care models.

## Methods

Realist synthesis is a systematic, iterative, theory-driven approach designed to make sense of diverse evidence about complex interventions applied in different settings [32–34]. The rationale for using a realist synthesis approach for this review is that interventions to promote shared decision making (SDM) in older people with complex health and care needs are likely to be multi-component and are contingent on the behaviours and choices of those delivering or receiving the care.

A realist synthesis assumes a ‘generative’ approach to causation, that is, “to infer a causal outcome (O) between two events (X and Y), one needs to understand the underlying mechanism (M) that connects them and the context (C) in which the relationship occurs. It is typically used to understand complex interventions that ‘often have multiple components (which interact in non-linear ways) and outcomes (some intended and some not) and long pathways to the desired outcome(s)” [32, 35]. Central to the realist review process is the development of programme theory, i.e. what a programme or intervention comprises and how it is expected to work. The review followed the Realist and Meta-narrative Evidence Syntheses: Evolving Standards (RAMESES) publication standards for realist syntheses [36]. A fuller version of the methods is published elsewhere [37].

The synthesis focuses on community dwelling older people with complex health and care needs, for example, people with frailty, multi-morbidity and long-term conditions. The rationale for focusing on this group is that they often use several health and social care services, their needs change over time and/or suddenly, sometimes with progressive loss of cognitive and/or physical function, a family carer is frequently involved in their care, and they are often at risk of exacerbation of their illness and death [17]. In addition, many find it difficult to navigate complicated and under-resourced services and are particularly vulnerable to fragmented care [38]. The focus was generally on those aged 65 years or over, although for certain groups (e.g. people from black and minority ethnic groups (BME), those with mental health problems) we included some younger participants (≥55 years) if the issues were similar. We used an iterative, stakeholder driven approach with three phases.

### Phase 1: Development of initial programme theory/ies

In Phase 1 we sought to develop candidate theories about why programmes that seek to promote SDM do, or do not, work. The starting point was systematic reviews of SDM and related topics (such as person-centred care). To identify reviews we searched PubMed and the Cochrane library using the following MESH terms: shared decision making, patient participation, patient decision making, decision support, decision aid, expert patient, proxy decision making, collaborative care, co-construction, coproduction and minimally disruptive medicine. These terms were combined with methodological search terms for systematic reviews. In addition, we undertook key word searches on Google Scholar for both reviews and primary studies and looked for relevant papers published by key authors in the area. We identified 39 systematic reviews and 35 primary studies. In addition, we undertook face to face or telephone interviews with stakeholders in England including user/patient representatives, health care professionals and commissioners/funders, and service providers in integrated care sites. We recruited 13 stakeholders rather than the 20 specified in the protocol. Interviews were conducted using realist principles [39] and participants were a convenience sample recruited for their known expertise in SDM and care of older people. The purpose of the stakeholder consultation was to explore key assumptions about what needs to be in place for effective SDM and identify relevant outcomes. Ethical approval was obtained from the University of Hertfordshire Health and Human Sciences Ethics Committee with delegated authority (ECDA), reference number HSK/SF/UH/02387.

The literature and stakeholder interviews were used to develop preliminary hypotheses in the form of five ‘If-Then’ statements (Table 1). These if-then statements, which helped to specify context and mechanism, were illustrated with supporting evidence from the interviews and literature. ‘If-Then’ statements are the identification of an intervention/activity linked to outcome(s), and contain references to contexts and mechanisms (though these may not be very explicit at this stage), and/or barriers and enablers (which can be both mechanism and context) [40]. The ‘If-Then’ statements helped to focus the process of taking ideas and assumptions about how interventions work and testing them against the evidence we found.

**Table 1:** Preliminary programme theory in the form of if-then statements

Title	If	then	Outcome
Reflecting patient and carer values	If health care professionals (HCPs) place less emphasis on ‘fixing people’ and more on patients’ goals, and emotional, cultural & cognitive needs	Patients and their carers will feel valued and listened to	Patient and their family carer feel involved in the decision and satisfied with the outcome
Preparing (patients and carers) for the SDM encounter	If older people with complex health and social care needs are supported to participate in SDM	Patients and their family carers will feel empowered	The patient and family carers are willing and able to participate in SDM
Sharing the communication of a decision	If HSCPs are familiar with each other’s expertise, roles and responsibilities, and systems facilitate communication between individuals	Professionals will work better together and are less likely to undermine each other	Once a decision has been made by the patient and a health care professional it will be shared across the MDT/agencies
Fake vs real SDM	If systems are organised to support and prioritise SDM	SDM is not just seen as a ‘tick box’ exercise by health care professionals	SDM is authentic not tokenistic
Reducing the workload (for patients and carers)	If HSCPs use appropriate SDM techniques to regularly discuss the clinical value and effectiveness of proposed treatments or interventions	This leads to reduction in inappropriate clinical activity	Reduced treatment burden

* = truncation symbol

The ‘If-Then’ statements were further developed through discussions at a workshop attended by eight members of the research team and consultation with the Project Advisory Group. The Advisory Group included experts in the field of older people’s health, primary care, patient involvement and realist methods, and experts by experience (Public Involvement representatives).

### Phase 2: Retrieval, review and synthesis

In Phase 2 we undertook systematic searches of the evidence to test and develop the theories identified in Phase 1. The focus of the review was on community dwelling older people with complex health and care needs, such as those with frailty, multi-morbidity, dementia. However, we also included other populations where the study was considered to offer opportunities for transferrable learning. Other inclusion criteria were as follows:

- Older people with complex health needs living in their own homes, in sheltered housing or extra care housing.
- Any intervention or strategy designed to promote the ongoing engagement of older people with complex health needs, and/or their family carers, in decision making relating to their health or social care needs (e.g. decision aids, physician or patient coaching, education or training, personalised care planning, joint goal setting). The focus was on complex decision making and personal goals rather than single issues (such as whether to have a hip replacement). This included:
  - ○ Interprofessional SDM where at least two health care professionals collaborate to achieve SDM with the patient and/or family carer either concurrently or sequentially [41]
  - ○ Studies that provide evidence relating to the implementation and uptake of interventions designed to promote SDM for older people with complex health needs.
- Published and unpublished studies of any design.

Data sources included: Medline (PubMed), SCOPUS, The Cochrane Database of Systematic Reviews, DARE (Database of Abstracts of Reviews of Effects), the HTA Database, NHS EED (NHS Economic Evaluation Database), Google and Google Scholar. The searches were designed to reflect the five ‘If-Then’ statements identified in Phase 1. Date limits and search terms used in PubMed can be seen in Table 2. In addition, we undertook lateral searching such as forward and backward citation tracking. The purpose of the searches was not to identify an exhaustive set of studies but rather to identify sufficient sources for building and testing our programme theory [42]. As is usual with a realist review, the process of identifying relevant information and deciding what to include was iterative involving tracking backwards and forwards between the literature and our review questions [43].

**Table 2:** Details of search terms – using PubMed as an example

Theory area & Search terms	
1. Reflecting patient and carer values	
((“shared decision making”) OR (“decision aid”) OR (“decision making”)) AND (((“goal setting”) OR (“person centred care”) OR (“person centered care”) OR (“personalised”) OR (“patient goals”) OR (“patient values”) OR (“patient preferences”) OR (personalised[Title] OR personalized[Title] OR (patient centred) AND Title OR (patient centered) AND Title OR (patient preference*) AND Title OR goals[Title] OR (goal setting) AND Title OR personalised[Title])) AND (old*[Title] OR aged[Title] OR elder*[Title] OR geriatric[Title] OR frail[Title] OR complex[Title] OR complex[Title] OR carer[Title] OR dementia[Title] OR alzheimer*[Title])) No date limits	
2. Preparing for the SDM encounter	
*Coaching/advocacy* (((“coaching”) OR (“advocacy”) OR (“advocate”) OR (advocate[Title/Abstract] OR advocacy[Title/Abstract] OR coach*[Title/Abstract]) OR (“coach”)) AND ((“shared decision making”) OR ((shared decision making) AND Title/Abstract OR SDM[Title/Abstract] OR decision[Title/Abstract]))) AND ((“frail elderly”) OR (“older person”) OR (“dementia”) OR (“elderly”) OR (old*[Title] OR elderly[Title] OR frail[Title] OR dementia[Title] OR alzheimer*[Title] OR aged[Title])) No date limits	
*Education/training* ((“shared decision making”) AND (education[Title] OR educate[Title] OR training[Title] OR guidance[Title] OR support[Title] OR information[Title] OR guide[Title] OR train[Title])) AND (old[Title] OR older[Title] OR elder*[Title] OR frail[Title] OR complex[Title] OR carer[Title] OR geriatric[Title] OR aged[Title] OR dementia[Title] OR alzheimer[Title]) ((“shared decision making”) AND (education[Title] OR educate[Title] OR training[Title] OR guidance[Title] OR support[Title] OR information[Title] OR guide[Title] OR train[Title])) AND (“primary care”) No date limits	
*SDM for hard to engage groups (e.g. those with depression)* ‘Shared decision making’ OR ‘decision aid’ (both MeSH) OR (coproduction[Title/Abstract] OR co-productive[Title/Abstract] OR partnership[Title/Abstract] OR co-production[Title/Abstract] OR co-production[Title/Abstract]) AND ‘depression’ OR ‘mental health’ OR ‘mental illness’ (Mesh) AND systematic review Filters: published in the last 5 years Shared decision making’ OR ‘decision aid’ (both MeSH) OR (coproduction[Title/Abstract] OR co-productive[Title/Abstract] OR partnership[Title/Abstract] OR co-production[Title/Abstract] OR co-production[Title/Abstract]) AND ‘depression’ OR ‘mental health’ OR ‘mental illness’ (Mesh) AND ((“frail elderly”) OR (“older person”) OR (“dementia”) OR (“elderly”) OR (old*[Title] OR elderly[Title] OR frail[Title] OR dementia[Title] OR alzheimer*[Title] OR aged[Title]))	
3. Sharing the communication of a decision	
*Interprofessional (limited to last 10 years)* (((“interprofessionalism”) OR (“interprofessional”) OR (“interdisciplinary”) OR (“multidisciplinary”) OR (“coordinated”) OR (“cross discipline”) OR (“inter disciplinary”) OR (“integrated”)) AND ((“shared decision making”) OR (“decision aid”) OR (“decision making”))) AND (old*[Title] OR aged[Title] OR elder*[Title] OR geriatric[Title] OR frail[Title] OR complex[Title] OR complex[Title] OR carer[Title] OR dementia[Title] OR alzheimer*[Title]) “relational coordination” OR “relational coproduction” AND (old*[Title] OR aged[Title] OR elder*[Title] OR geriatric[Title] OR frail[Title] OR complex[Title] OR complex[Title] OR carer[Title] OR dementia[Title] OR alzheimer*[Title]) No date limits	
*Relational competence* Relational competence AND (promote[Title/Abstract] OR promotion[Title/Abstract] OR train*[Title/Abstract] OR increase[Title/Abstract] OR intervention[Title/Abstract] OR programme[Title/Abstract]) AND general OR community OR primary No date limits	
4. Fake vs real SDM	
Draws on searches run for other theory areas. Incentive (ti/ab) OR incentives (ti/ab) OR incentivisation [TI/AB] OR incentivization [TI/AB] AND “shared decision making” (MESH) No date limits	
5. Reducing the workload (for patients and carers)	
(((“minimally disruptive medicine”) OR (“caregiver burden”) OR (“carer burden”) OR (“patient burden”) OR (“treatment burden”) OR (“quality of life”) OR (appropriate[Title] OR inappropriate[Title])) AND (“shared decision making”)) AND (old[Title/Abstract] OR older[Title/Abstract] OR aged[Title/Abstract] OR elderly[Title/Abstract] OR frail[Title/Abstract] OR carer[Title/Abstract] OR complex[Title/Abstract] OR geriatric[Title/Abstract] OR dementia[Title/Abstract] OR Alzheimer[Title/Abstract]). Limited to last 5 years	

### Selection and appraisal of documents

Search results were downloaded into bibliographic software. Two reviewers independently screened titles and abstracts identified by electronic search and applied the selection criteria to potentially relevant full-text papers. Decisions on inclusion were recorded in an excel spreadsheet. Consistent with a realist review approach, items were assessed for inclusion on the basis of whether they were considered ‘good enough and relevant enough’ [44, 45]. This was an iterative process that involved discussion between team members. Good enough was based on the reviewers’ own assessment of the quality of evidence, for example was it considered to be of a sufficient standard for the type of research, and whether the claims made were considered trustworthy. Relevance related to whether the authors provided sufficient descriptive detail and/or theoretical discussion to contribute to the theories generated in Phase 1. Studies considered by the team to be poorly executed could still be included if the study was considered to contribute to understanding about how a programme was thought to work.

### Data extraction and synthesis

A data extraction form was developed, piloted on five records and further refined as necessary. Once the final fields for data extraction were agreed, an electronic version was created in MS Access. The data extraction form included fields relating to study aims, design and methods; the types of participants (e.g. older people, people with long-term health conditions, HSCPs); outcomes; information relevant to the theory areas; and emerging CMOs. Data were extracted by one reviewer with 20% checked by a second reviewer. PDFs in Mendeley were also annotated and relevant sections highlighted. Data in a realist sense are not just restricted to the study results or outcomes measured but also include author explanations and discussions, which can provide a rich source of ‘data’ that makes explicit how an intervention was thought to work or not. The query feature in the ACCESS database was used to create tables to facilitate the identification of prominent recurrent patterns of contexts and outcomes (demi-regularities) in the data and the possible means (mechanisms) by which they occurred [46]. This deliberative and iterative process enabled iteration from plausible explanations to the uncovering of potential context-mechanism-outcome (CMO) configurations.

### Phase 3: Testing and refining of programme theory

In Phase 3, we tested the programme theory via interviews with 11 stakeholders and through discussions with the research team and Project Advisory Group. An interview schedule, based on the four CMOs, was used to elicit stakeholders’ views on their meaningfulness, both from practice and service user/carer perspectives. The interview data were used to test the CMOs.

## Results

**Fig. 1:**
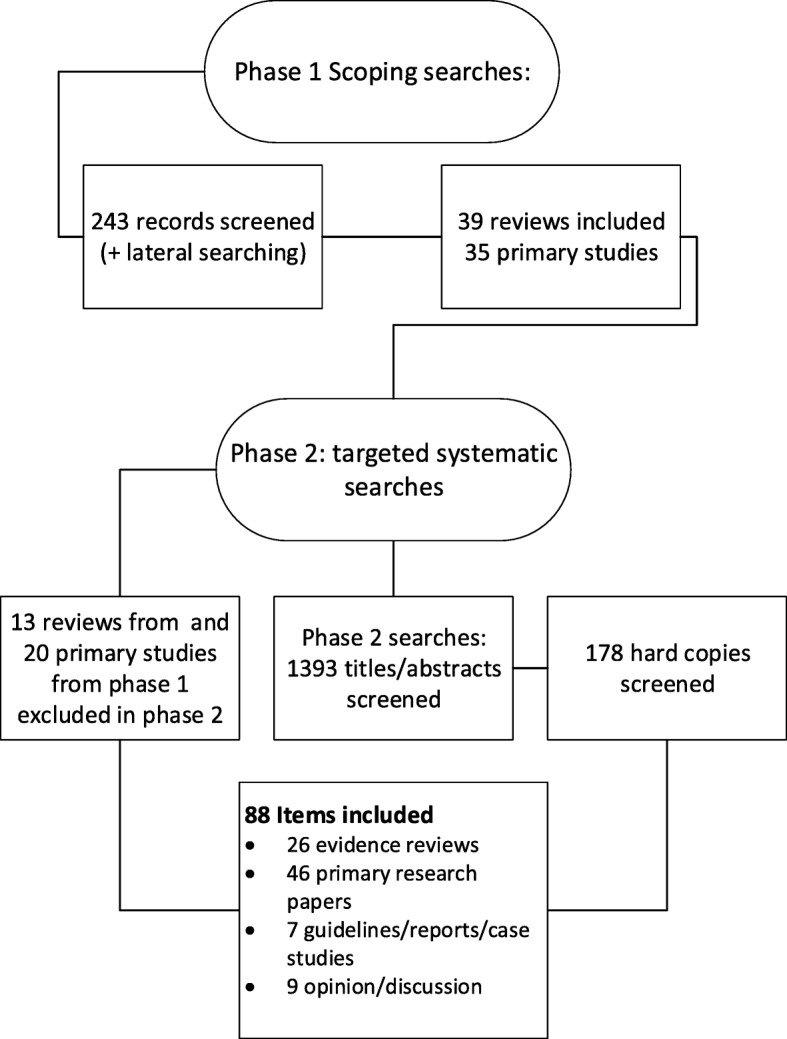
Flow chart summarising study identification

We included 88 items that included 26 evidence reviews, [3, 30, 31, 47–69] 46 primary research studies, [41, 70–116] seven guidelines, cases studies or reports, [8, 14, 117–121] and nine discussion/opinion papers [79, 122–129]. Of the 46 primary research papers, 25 were qualitative studies, five were RCTs and the rest included a variety of study designs. Of the evidence reviews, 20 were systematic reviews, [3, 30, 31, 47–50, 52–56, 58, 60, 62–66, 69] five were literature reviews, [51, 57, 61, 67, 68] and one was a realist synthesis [59]. The study selection process can be seen in Fig. 1. Thirty-three papers from Phase 1 were excluded at Phase 2 because they were not considered to be of high enough rigour or relevance.

The included literature either focused specifically on SDM or on aspects of care, such as person-centred care or personalised care planning, in which SDM plays an essential if not specified part with the patient or their proxy. We categorised the included reviews and other items according to the focus of the paper. The numbers in each category can be seen in Table 3. Twenty-five primary studies and four systematic reviews focused on older people or those with complex health and care needs. Of those 19 focused on older people or had a population with a mean or median age over 65, nine specified that people had multi-morbidities and 11 that they had long-term conditions.

**Table 3:** Overview of study focus

Category	Number of primary studies/items*	Systematic reviews
Professionals views on SDM	10	2
Interprofessional SDM	13	1
Use of Patient decision aids/tools	13	10
Patient engagement in SDM	17	7
Influences on SDM	33	4
Education/training HSCPs	13	4
Patient/carer views/preferences/goals	30	9

* = truncation symbol

Sixteen reviews were evaluating an intervention, such as decision aids or tools, coaching, and interventions to increase or promote the adoption of SDM amongst health care professionals. Nineteen of the other papers described or evaluated an intervention. Interventions included care planning, training and education for professionals, the use of decision aids or integrated/collaborative care practices that involved SDM. More details of the reviews can be seen in Additional file 1 and of other items (e.g. primary studies) in Additional file 2.

The theory development, refinement and testing process led to the development of four context-mechanism-outcome (CMO) configurations (see Table 4). Together, these explanations or hypotheses constitute a programme theory about ‘what works’ (or ‘what might work’) to facilitate shared decision-making (SDM) for older people with multiple health and care needs or conditions, and how they might be applied within models of integrated working. Supporting evidence from the stakeholder interviews can be seen in Table 5.

**Table 4:** Overview of four Context-Mechanism-Outcome configurations that make up the programme theory

Programme theory	Supporting evidence
*CMO1 Reflecting patient and carer values*: Systems that enable HSCPs to develop relationships with patients and carers, and with each other, and that allow them to understand and assess individual needs and patient and carer capacity to access and use care, will activate trust and engagement leading to better outcomes for patients and carers.	[30, 31, 47, 50, 51, 53, 56, 59, 63, 65, 67], [41, 72–74, 77, 80–83, 87, 90, 91, 93, 94, 96–98, 100–106, 109, 110, 113, 117–121, 124, 128]
CMO2: Systems that are organised to support and prioritise SDM will lead to HSCPs feeling supported (and equipped) to engage in SDM resulting in SDM becoming part of the culture of care.	[30, 31, 53, 56] [8, 14, 80–82, 85, 86, 96, 106, 121] [74, 83, 87, 103, 104, 110, 122, 124, 128] See
CMO3: People with complex health and care needs, and their family carers, are likely to need support, such as appropriate decision tools, and space and time to ask questions and discuss options, in order for them to be willing and able to participate in SDM.	[48, 49, 54, 58, 61, 62, 68, 69] [8, 14, 70, 84–86, 92, 95, 96, 107, 108, 113, 117, 118, 121] [83, 89, 90, 94, 125] [75, 76, 99, 129] [63]
*CMO 4: SDM as part of a wider cultural change* (e.g. family centred approaches, changes in power dynamics and patients and carers taking (or sharing) responsibility for their health and the decisions which affect them), triggers the development of a shared expectation of (and familiarity with) SDM amongst patients, carers and HSCPs leading to improved patient outcomes.	[60, 62, 65, 68] [49] [50, 53] [8, 14, 82, 87, 96, 121, 122]

**Table 5:** Examples of supporting evidence from stakeholder interviews

CMO1: Reflecting patient and carer values	
Patient capacity to access and use care	“It (refers to SDM) makes it easier to avoid situations where people either don’t understand what the medication that they’re being prescribed is for, when to take it, how to adjust it with other medication that they may be on, and so on. It can lead to...to a plan which is grounded in shared expectation.” SH06
Interprofessional approaches to PCC	“So whether someone is seeing one clinician all of the time and over time making a number of decisions, or if they’re being seen in five different clinics over the course of whatever, the fact that that ethos of person-centeredness is embedded across that, you know, and their information shared and they build on it…” SH15
Patient feels involved and engaged	“…when you’re offered an opportunity to discuss your own care you feel as proud as anything…” SH02a
Patient centred approaches	“…he then saw where we were going with his treatment...he was an active participant whereas before he’d been very much, “No, I don’t want to do this, I don’t want to do that”.” SH10
Goal setting	“I think the Year of Care Programme is another example of that, which was started in diabetes which focused on, you know, care and support planning, that’s how they framed it but essentially is about people making decisions together about what matters to them, setting their goals and then making decisions about what treatments and other things will support that.” SH15
Adherence	“From the clinicians’ point of view, the benefits (refers to SDM) are that there’s an increased likelihood of adherence to clinical plans and to prescribed medicines. It leads to better use of resources…” SH06
Feeling valued	“The consultant even phoned me at home and said, “This is what’s happening, this is what we need to do,” so I was fully involved when my mum, you know, lost capacity for those few days, and I felt very valued…” SH07a
Continuity – individual and system based	“…when you’re talking about allowing them to develop the relationship, are we thinking about continuity over time or are we thinking that actually we’ve just got a system that supports person-centred care and that values that as part of any consultation.” SH15
CMO2: Systems to support SDM	
Risk	“…on Monday that I had, a patient who has quite significant dementia who’s in her 90s, and there’s a lot of sort of indecisions about where, whether she should be at home, whether she should be in a care home or supported accommodation. There are clearly, you know, now some risk issues by her remaining at home on her own, but, you know, after a lot of sort of decision and discussion, I guess, you know, the decision was that it’s best, that’s where she was best to be even though we were all expecting some degree of risk…” SH03
Risk	“…she said, “No, I don’t want to take any tablets, thank you very much. I know the risk.” That’s fine…” SH10
System based approaches	“…we work with clinical colleagues here who do that [send results to patients before a consultation] in diabetes a lot and that works well and it just seems to make sense doesn’t it? You don’t go along to your bank manager and have a discussion about your bank account without knowing what your balance is…” SH20
System based approaches	“crucially, the patient is able to see the outcomes of all of those tests in advance of their care planning discussion, which means that they’re able to think about what that means for them, and a good care planning template will have on the front some free text boxes which ask questions like, “What’s most important to you to discuss in the care planning conversation?” “Have there been any changes since we last spoke that you’d like to raise?” “Do you have any questions?” and so on, which means that the conversation, alongside taking into account the person’s clinical needs, also gives an invitation, I suppose, to the person, to feed in the other aspects of their life…” SH06
CMO3: Preparing for the SDM encounter	
Family involvement	“So if you’re doing a care planning meeting with an older adult with multiple conditions that you give them a chance to have a think about it, often with their family member as well.” SH10
Choice	“So it’s not about what people want, it’s about where there are options, understanding, so the patient and carers need to understand what the options are, you know, what the risks, the benefits, the consequences of the different options are and they need to understand what’s important to them in deciding between them.” SH20
Asking questions	“…the provision of really high-quality information for people, we know that that makes a really significant contribution for people, increasing their confidence, potentially increasing their levels of literacy, in terms of their understanding of their condition and how it impacts on their life, but also being more confident to ask the questions that they need to from their clinicians, and to offering their own perspective…” SH06
Asking questions	“… there was a video for patients and there was the “ask three questions”, materials that were used throughout...showing the video on, you know, in the waiting room in the GP surgery or whatever, that actually that had little or no impact on increasing the likelihood of patients asking those questions of their healthcare professional, but where it did have an impact is that it meant that the clinicians were much more likely to prompt patients around those questions.” SH15
Medical authority	“…my parents, because they were both in their 90s when they died, they would assume somebody with, anyone medical had authority.” SH17
CMO4: SDM as part of a wider culture change	
Power differentials	“…the power differentials are one of the bigger barriers to shared decision making and so it is about recognising, it’s a fairly simple thing to say, but recognising there are two experts, that the clinician who understands the options and the risks, the benefits, the consequences and so forth and the patient who understands what’s important to them.” SH20
Change	“…how we have always framed, you know, our shared decision making and our self-management work is that this was part of essentially a transformational change…” SH15
Wider change	“…there is no intervention that creates culture change, whatever it is, but it’s absolutely right that it has to happen and that happens because all sorts of different things get aligned if you like but that takes time and it has all the issues that you’ve already talked about around systems, skills, attitudes, education, training, patient roles, all of those things need to be aligned…” SH20
Changing attitudes	“Yeah, so I think some of it will be attitude changes, I think some of it will be cultural. I think some of it will come, so we are seeing shifts within new care models to, you know.” SH06
Culture	“…really good quality, shared decision making, comes largely from the culture, and through communication and between clinical teams and people…” SH06
Patient responsibility	“…changing attitudes and experiences of patients can be at times as much a barrier to shared decision making as the attitudes of the clinicians.” SH20
Attitudes	“…there are some really important attitudinal underpinnings that need to be addressed before you can even do the skills training…” SH20

### CMO1: Reflecting patient and carer values

#### Understanding the needs and priorities of service users/patients/carers

Considering patients’ and, where appropriate, family carers’ preferences and values is seen as key to the decision-making process [51, 59, 67, 102, 103, 124]. Systematic review evidence suggests that interventions to promote a patient-centred approach in clinical consultations can have a positive effect on a range of measures although the impacts on satisfaction, behaviour and health status were mixed [56]. Despite this, individual needs and circumstances of patients and their family carers are frequently not taken into account [53, 74, 96, 98]. Reasons for information sharing difficulties, and goal divergence include health care professionals having difficulty identifying patient preferences, [47, 50] differences in the way patients and clinicians interpret and frame the patient’s health problems [94, 110] and clinicians being reluctant to engage in SDM when the patient’s preferences are not in line with clinical guidelines [106] or when there are concerns about safety or cognitive function [98].

#### Developing relationships

Achieving collaborative approaches to care, such as SDM, depends on building a good relationship in the clinical encounter [59, 74, 83, 90, 97, 101, 103, 104, 109, 115, 128]. This impacts on patient and carer perceptions of the quality of care, [55, 80, 82] and may improve adherence to medical treatments [55]. Increased trust was associated with longer consultations, physician verbal behaviour (such as exploring the impact of the condition or illness on the patient) [130] and continuity of care. [47, 59, 93]. The importance of ongoing relationships and the ability to reassess changing priorities were highlighted in several studies [47, 103]. This was particularly important for people with complex needs or dementia as ‘the dominant chronic illness shifts over time as conditions and treatments change, and re-prioritization occurs’ [51] and decision-making responsibility may shift over time, from the person with dementia to the family carer [91].

#### Interprofessional working

Partnership working between different health and care professionals was seen as key to decision making for older adults with complex needs [73, 78, 91, 97]. Facilitators of interprofessional working include a history of working together, mutual knowledge and understanding of disciplinary roles, trust and respect, a shared understanding of SDM, and effective communication between individuals (including different health and social care practitioners and patients & carers) [41, 64, 80, 100, 120]. However, few studies addressed an interprofessional approach to SDM, with most studies targeting a single professional group [41, 65].

#### Patient and carer outcomes

Systematic reviews suggest that interventions to promote SDM may lead to patients and carers feeling more involved in decision making [63, 67]. There is also evidence of improved quality of life and reduced depression in carers, [67] and improved affective cognitive outcomes for patients, such as increased satisfaction and reduced decisional conflict [47]. These impacts (particularly on patient satisfaction) are echoed in many of the other studies we accessed. There is also some evidence that SDM leads to better treatment adherence [118]. There is little evidence, however, to suggest that there is an association between empirical measures of SDM and health outcomes [47].

### CMO2: Systems to support SDM

Studies support a link between organisational ‘buy-in’ (e.g. identifying SDM as an organisational priority) and an increase in health and social care practitioner engagement with, and prioritisation of, SDM. However, whilst SDM is a core part of policy in many countries, including the UK, [131] at a service level, systems are not in place to incentivise or appropriately reward patient-centred practice and SDM [96, 128]. Furthermore, for older people with complex conditions SDM is hindered by the risk and uncertainty associated with complex conditions and by systems and structures that block communication between patients and the different professional groups involved in their care [96]. The literature outlines several system based approaches to improve SDM. For example, preparatory work to support the patient’s involvement in decision making, e.g. an initial appointment with a nurse or health care assistant before a meeting with the GP [14], longer appointments, [85, 86] and annual reviews which include monitoring for all chronic conditions [14, 110, 121]. However, little data on patient outcomes are available.

The need for enhanced communication skills among clinicians was a common theme across the papers [30, 47, 53, 56, 59, 87]. This included the ability to address with patients the uncertainty involved in many medical and care decisions [103, 122]. Several studies reported that training to promote person-centred approaches and SDM had positive impacts on SDM skills and engagement [56, 65, 85, 86]; there was less evidence of changes in patient focused outcomes [85, 86, 96]. A UK based study reported that interactive skills training workshops based on a SDM model helped build coherence, improving skills, and promoting positive attitudes. It was also considered important that clinical teams were able to develop a shared understanding of how SDM might differ from their current practice [96].

### CMO3: Preparing for the SDM encounter

#### Decision support

Much of the literature on preparing patients and carers relates to the use of patient decision aids; tools designed to help people participate in decision making about health care options [132]. Systematic reviews provide good evidence that patient decision aids can have a positive impact on patient knowledge, decisional conflict, informed choice, participation in SDM and decision self-efficacy, [3, 48, 49, 54, 62, 69] including for those who are socially disadvantaged [48]. Potential mechanisms relating to the likely benefits of decision aids include patients becoming more engaged, [48] developing greater decisional self-efficacy, [48] feeling more involved in decisions, [85, 86] and increased mutuality [90]. However, the reviews provide little evidence that decision aids improve health outcomes or patient adherence.

Older age, depressive symptoms and difficulties with activities of daily living are associated with decreased patient activation [89]. There is some evidence that decision aids can enhance older adults’ participation in SDM [54, 69]. However, most evidence relates to younger older people (70 years and under) rather than the oldest old (80+) and most tools are not tailored to the needs of people with multi-morbidity [69]. Moreover, there is unlikely to ever be a patient decision aid for every decision, not all patients will find them acceptable or helpful [96], and they may not address the entry level factors to SDM, such as subjective norms and patients’ roles [62, 90]. There is some evidence that the use of coaching or guidance may support patients in the process of thinking about a decision and in communicating their values and preferences with others [68, 70, 92]. The mechanisms inferred from these papers are that improving patients’ deliberation and communication skills will lead to empowerment and thus patients will feel better supported. However, the impact on other outcomes, such as participation in decision making or satisfaction with option chosen, is mixed [68].

#### Permission/space to discuss option

Key to CMO 3 is that SDM is undertaken in a context where patients and their families can discuss the value and effectiveness of proposed treatments without feeling judged. Longer consultations are linked to greater patient satisfaction and improved SDM, [14, 53, 74, 83, 85, 101, 104, 113] which is likely to be related to the opportunity for patients to ask questions, and feel listened to [83, 101] and respected [97, 109]. However, clinicians’ attitudes may act as a barrier to SDM with older people feeling unable to make their needs heard [76] or reluctant or unable to discuss relevant context or preferences during a consultation [75, 76]. Moreover older people may not always be aware that there is a choice to be made [76]. Research has underscored the importance of family-centred approaches for older people with complex needs [18, 133]. However, similar to a realist review on engaging older adults in healthcare decision making [59], we found few studies that considered the involvement of family members and friends in SDM.

### CMO 4: SDM as part of a wider culture change

#### Time and resources

The programme theory outlined in CMOs 1–3 outlines many barriers to SDM and it is clear that relying on individual clinicians or patients to implement SDM without system-based support is unlikely to be successful or sustainable [60, 62, 65]. Several included papers described system-based changes that involve person-centred, integrated approaches to people with long-term conditions, [8, 14, 82, 121] of which SDM is an integral part. These initiatives reported increased staff and patient satisfaction [8, 14, 121] although the impact on clinical outcomes is not clear. One report suggested that changing patient and professional habits may need a number of care planning cycles [121]. This is reflected in our programme theory which argues that familiarity and a shared expectation of new ways of working (which include SDM) are likely to take time to develop.

#### Patient activation or engagement

The willingness or ability of patients to participate in SDM is a key contextual factor in our programme theory (see also CMO3). This was supported by the literature, [53] and underscored by our interviews with stakeholders. In general, the consensus from the literature is that although the majority of older people would wish to be involved in decision-making in practice they are often not encouraged, or enabled, to participate in SDM [50, 62, 96]. Reasons for this include limited time, poor continuity of care, environmental conditions, organisational inertia, a biomedical focus, concern about disruption to routines, clinicians’ belief that they are already practicing SDM, and power imbalances [60, 62, 87, 122, 134]. Whilst many SDM initiatives involve giving patients more information, this alone is not enough. Patients need knowledge and power to participate in SDM [62, 135]. A systematic review of patient reported barriers and facilitators to SDM suggested that power may be linked to perceptions of permission to participate in decision making, perceived influence on decision making, confidence in own knowledge and self-efficacy in SDM [62].

## Discussion

### Summary of the findings

**Fig. 2:**
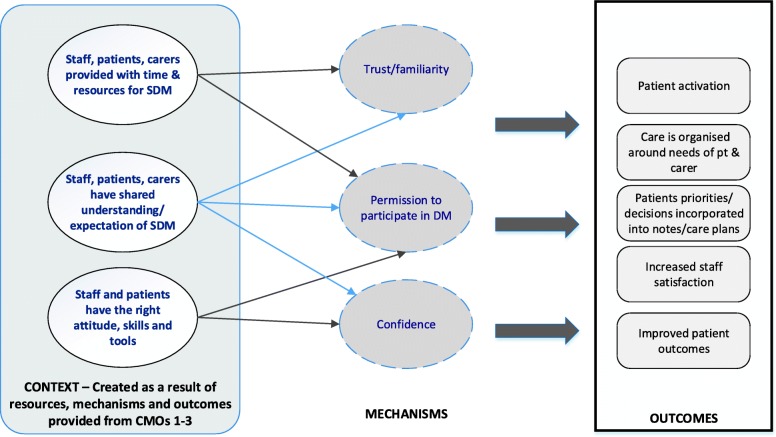
Summary of programme theory: the figure depicts how the context is created as result of the resources, mechanisms and outcomes provided by CMOs 1–3

We have developed an explanatory account of what SDM should look like for older people with complex health and care needs (see Fig. 2). Our theory suggests that programmes that are likely to be successful in fostering SDM between older people with complex needs, their family carers and service providers are those that create trust between those involved, that allow older people to feel that they are respected and understood, that are accessible to older people and that engender confidence to engage in SDM. Confidence is likely to take time to develop as, we suggest, it is related to the development of a shared understanding and expectation of SDM between patients and HSCPs. The cultural shift that is needed to embed SDM in practice may require new ways of working for professionals and a shift away from a biomedical focus to a more person-centred ethos that goes beyond the individual patient encounter. To achieve this, health care professionals are likely to need support, both in terms of the way services are organised and delivered and in terms of their own continuing professional development. Older people with complex needs and their family carers may also need support to engage in SDM, which includes interventions that are adapted to their needs (in terms of literacy, health literacy and computer literacy, among other things). How this support might best be provided needs to be further explored, although face to face interactions and ongoing patient-professional relationships are clearly key.

### Strengths and weaknesses of the study

One of the main limitations of this review is the lack of evidence around interventions to promote SDM in older people with complex health and care needs. The lack of evidence is compounded by little evidence around SDM in integrated care teams. However, in realist methodology, the unit of analysis is the programme theory, or underpinning mechanism of action, rather than the intervention [43]. This meant we were able to draw on a wider literature that provided opportunities for transferable learning, for example studies involving people with long-term conditions or mental health problems. This enabled us to develop a programme theory which can inform initiatives to promote SDM for older people with complex needs. Whilst our searches were systematic the broad nature of our inclusion criteria means that we may have missed potentially relevant literature. However, the nature of realist methodology means that there is not a finite set of relevant papers to be found. Instead the reviewer is able to take a more purposive approach to sampling that aims to identify sufficient sources for theory building and testing rather than identify an exhaustive set of documents [42, 43].

### Strengths and weaknesses in relation to other studies

Person-centred approaches to health and care and considering each patient’s preferences and values are central to the SDM process [12]. For older people with complex needs eliciting preferences is likely to involve regularly revisiting decisions because the dominant illness, and priorities, may shift over time [51, 91]. However, the evidence suggests that doctors are better at recognising and discussing options than eliciting patient preferences (see CMO 1). This may reflect the fact that different health and social care practitioners conceptualise person-centred care in different ways [136, 137]. A review of literature on person-centred care suggests that whilst the nursing literature tends to focus more on respecting patients’ values and beliefs in promoting person-centred care, the medical literature has devoted more attention to understanding the nature of the informed decision-making process between the doctor and the patient [136]. What is not explored in the literature is whether integrated care and interprofessional working might enable different members of the multi-disciplinary team to draw on the skills of others in order to promote effective person-centred approaches to SDM.

### Meaning of the study

The quality of individual clinicians’ communication skills, and their ability to foster trusting relationships with older people and their families, is fundamental to SDM. SDM education and training should be focused on all members of the multidisciplinary team and not just doctors or lead clinicians. It should be part of undergraduate training programmes but also part of ongoing professional development and should include exploring what matters to patients and how to elicit their goals and priorities. In addition, there is also a need for systems that foster continuity of care. Continuity can be achieved through an ongoing relationship with one clinician (relationship continuity) or a system based approach that develops ways of working whereby the patient is linked to multiple professionals (management and informational continuity) [138–140]. The evidence would suggest that both need to be in place. Informational continuity is, however, often hindered by electronic systems not set up to record information relating to patient preferences and goals [110]. The evidence highlights key contextual factors to facilitate SDM for older people, including consultation length, clinicians’ communication skills, and whether it is possible to create a culture that allows people to ask questions without feeling judged. A culture that allows people time to ask questions and to discuss options, and staff with positive attitudes towards SDM are likely to be more important than decision support tools for older people with complex health and care needs. These resources are likely to lead to an increased ability and willingness to engage in SDM through mechanisms such as feeling respected and understood.

### Unanswered questions and future research

Evidence from stakeholders and from the literature suggests that older people with complex and competing health and care demands (and where depression is a common comorbidity) may need considerable support to enable them to engage in SDM. This can be exacerbated by factors such as deprivation, low health literacy or cognitive impairment. There is a need for more work to specifically focus on older people with complex needs, for example, more research looking at what is happening in SDM conversations involving older people with complex needs, how patient decision aids are being used and to what effect? More research is needed on family-centred approaches to SDM. For example, what is the impact of making it the default option (with consent from the older person) to involve designated family members in consultations and discussions about treatment options? In addition, whilst models for health care delivery are moving towards a more interprofessional healthcare team-based approach, [24] most evidence concerns decision making involving a single doctor and a patient, and there is a lack of studies addressing interprofessional approaches to SDM [65]. For interprofessional SDM to work the development and involvement of all staff are important [8, 100].

## Conclusions

Models of SDM for older people with complex health and care needs should move away from thinking about SDM purely in terms of one doctor/patient encounter. Rather SDM should be conceptualised as a series of conversations that each patient, and their family carers, may have with a variety of different health and care professionals. Such an approach relies on continuity of care fostered through good relationships between practitioners and patients, and systems that facilitate the communication of information, including that about patient goals and preferences, between different health and care professionals. The literature on SDM involving older people or those with complex needs is largely qualitative or descriptive and there are very few evaluations of interventions specifically designed to promote SDM with this group, and with their family carers. This review suggests there is need for further work to establish how organisational structures can be better aligned to the needs of older people with complex needs. This includes a need to define and evaluate the contribution that different members of the health and care team can make to SDM for older people with complex health and care needs.

## Additional files

Additional file 1: Table summarising details of included systematic reviews. (DOCX 21 kb)

Additional file 2: Table summarising details of included primary studies. (DOCX 79 kb)

## Abbreviations

CMO: Context-Mechanism-Outcome
HSCP: Health and Social Care Practitioner
PDA: Patient Decision Aid
SDM: Shared Decision Making
