# Supplementary material for: Supporting shared decision making for older people with multiple health and social care needs: a realist synthesis
Source: BMC Geriatr. 2018 Jul 18;18:165. doi: 10.1186/s12877-018-0853-9 (PMC6052575; doi:10.1186/s12877-018-0853-9)
Supplement: Supplementary file 1 — Table summarising details of included systematic reviews. (DOCX 21 kb) [file 12877_2018_853_MOESM1_ESM.docx]

Table summarising details of included systematic reviews

|  | | | | | |
| --- | --- | --- | --- | --- | --- |
| **Author and year** | **Aim/purpose** | **Design/method** | **Description of intervention** | **Sample size** | **Type of participants** |
| Austin 2015 | Identify decision tools for serious illness & summarise effect on outcomes and accessibility for clinicians | Systematic review  Each tool evaluated for its effect on patient outcomes and accessibility. | Decision aids - most are designed to be used prior to the consultation | 17 RCTs | Adults living with advanced or life-limiting diseases including 2 on older people and 4 on dementia |
| Belanger 2011 | Synthesize knowledge about the process of SDM in palliative care | Mixed methods SR Thematic analysis, conceptual mapping, and critical reflection. | NA | 37 studies (both quantitative & qualitative) | Palliative patients and families |
| Bratzke 2015 | Understand process of priority setting & DM amongst adults with multi morbidity | Narrative literature review | NA (but focused on studies of self-management) | 13 (mostly qualitative) | Adults with multimorbidity |
| Clayman 2016 | Assess the extent to which pt participation in DM is associated with measured patient outcomes. | Systematic review | Interventions to promote patient participation in DM | 116 studies | HCPs and a range of different patient groups and ages |
| Couet 2015 | Observe extent to which HCPs involve patients in DM across a range of clinical contexts | Systematic review of studies that had used the OPTION tool | use of the OPTION tool to facilitate SDM | 33 studies | HCPs and a range of different patient groups and ages |
| Coulter 2015 | Assess the effects of personalised care planning (PCP) for adults with LTCs compared to usual care | Systematic review of RCTs | Forms of care in which active involvement of pts in treatment and management decisions is explicitly attempted or achieved. | 19 studies involving a total of 10,856 participants. | Adults with long-term health conditions |
| Coylewright 2014 | Compare the use of DAs vs. usual care in consultations addressing DM, chest pain, osteoporosis or MI. | Patient-level meta-analysis of DA versus usual care comprising | Each RCT compared the use of a DA with UC. | 7 RCTs with 771 encounters between pts & | Adults expected to make a choice about a healthcare treatment or management strategy |
| Doyle 2013 | Explore evidence on the links between patient experience and clinical safety and effectiveness outcomes | Systematic review | NA - Identified +ve associations (where a better patient experience is associated with safer or more effective care), -ve associations and no associations | 55 studies | Varies between studies |
| Durand 2014 | Evaluate the impact of SDM interventions on disadvantaged groups and health inequalities | Systematic review and meta-analysis | Interventions or strategies designed to engage disadvantaged patients in medical decision-making and/or facilitate SDM | 19 studies, 10 were pooled in a meta-analysis | socially disadvantaged in respect of: 1) poverty/socioeconomic status; 2) ethnic minority status; 3) education/literacy level 4) geographical location |
| Dwamena 2012 | Assess the effects of interventions for health care providers that aim to promote patient-centred approaches in clinical consultations | Systematic review with meta-analysis | Interventions for healthcare providers that promote patient-centred care in clinical consultations | 43 RCTs | HCPs including those in training.  Patients were predominantly adults with general medical problems |
| Dy 2012 | Analyse key elements relevant to quality and complex, shared medical decision-making. | Literature review identifying key concepts relevant to SDM | NA | NA | Unclear |
| Edwards 2009 | Identify external influences on information exchange and SDM in healthcare consultations & conceptualise how information is used | Systematic review - (meta-data-analysis, meta-theory, meta-method, and meta-synthesis) | NA | 7 papers - Qualitative | HCPs and variety of patient groups |
| Elliot 2016 | Review available knowledge on engagement in healthcare decision making with a focus on older patients and their caregivers. | Realist synthesis | Interventions to engage older adults in health care decision making | 213 papers | Older people |
| Elwyn 2013 | Investigate the success of strategies to implement patient targeted decision support interventions into routine settings. | Systematic review with narrative synthesis | Focused on a)brief tools for face to face encounters b) more extensive tools such as booklets, videos, DVDs or websites | 17 studies included | Implementation was studied in primary and secondary care settings often involving multiple professions |
| Fagerlin 2013 | Summarize the current state of theory and evidence about the role of values clarification methods in DAs | Evidence review and summary | Value clarification in DAs | 61 studies (quant and qual0 | variety of patient groups |
| Joseph-Williams 2014 | Identify pt-reported barriers/ facilitators to SDM & develop a taxonomy of pt-reported barriers | Systematic review and thematic synthesis. | NA | 44 studies | variety of patient groups |
| Land 2017 | Map decision making communication practices relevant to health- care outcomes in face- to- face interactions | Systematic review of conversation analysis. | NA | 28 papers | People consulting a HCP |
| Legare 2008 | Identify barriers and facilitators to implementing SDM in clinical practice as perceived by HCPs | Systematic review with content analysis | NA | 38 papers | The vast majority of participants (n = 3231) were physicians (89%). |
| Legare 2012 | Evaluate patients' perceptions of the effectiveness of interventions designed to increase HCPs' use of SDM in routine clinical practice | Systematic review of quantitative studies | Included educational material; educational meetings; audit & feedback; reminders; and patient- mediated initiatives (e.g. DAs). |  | HCPs |
| Legare 2014 | Determine the effectiveness of interventions to improve HCPs adoption of SDM | Systematic review and meta-analysis | Interventions to improve healthcare professionals’ adoption of SDM | 38 RCTs, 1 non-randomised study | HCPs and patients. Most common clinical conditions included cancer and CVD. |
| Miller et al 2016 | Explore involvement of persons with dementia and their family carers in SDM | Literature review | NA - studies decision making processes | 33 papers were included in the review | No specific details given |
| Shay 2015 | Identify under what measurement perspectives SDM is associated with which types of patient outcomes | Systematic review | SDM studies that empirically measured the decision-making process with an SDM measurement tool in the context of a patient- clinician interaction and evaluated the relationship between SDM and health outcome/s | 39 studies | Patient groups not specified |
| Sinnott 2013 | Explore GPs’ experiences of clinical management of multimorbidity | Systematic review of qualitative studies | NA | 10 studies including 275 GPs | GPs |
| Stacey 2013 | To: define the concepts “coaching” and “guidance”; and summarise current theoretical and empirical insights into the roles played by coaching/guidance in the context of PtDAs | Narrative review (not described as a SR) | Coaching and guidance for SDM | N/A | N/A |
| Stacey 2014 | Assess the effects of DAs for people facing treatment or screening decisions | Systematic review & meta-analysis | Decision Aids | 115 studies | All patient groups (most look at specific health decisions such as screening options) |
| van Weert 2016 | Evaluate the efficacy of DA for older adults facing treatment, screening or care decisions | Systematic review of RCTs & CCTs | Decision aids vs usual care | Included 22 papers | Older people or their primary informal carers who have to make a health care/treatment decision. |
