# Supplementary material for: Supporting shared decision making for older people with multiple health and social care needs: a realist synthesis
Source: BMC Geriatr. 2018 Jul 18;18:165. doi: 10.1186/s12877-018-0853-9 (PMC6052575; doi:10.1186/s12877-018-0853-9)
Supplement: Supplementary file 2 — Table summarising details of included primary studies. (DOCX 79 kb) [file 12877_2018_853_MOESM2_ESM.docx]

Table summarising details of included primary studies

| **Author and year** | **If research give type** | **Description of intervention** | **Participants** | **Setting** | **Main focus** |
| --- | --- | --- | --- | --- | --- |
| Agoritas, T. et al 2015 ^80^ | Discussion | NA | General population | Applies to all healthcare settings | PDAs |
| Barrett 2016 ^175^ | Discussion | NA | Health care professionals | Community | communicating risk (re statins) |
| Belkora 2008 ^124^ | Before/after | Pre-consultation coaching | People with cancer | Community | Identifying pt goals |
| Berger 2015 ^174^ | Discussion | NA | Health care professionals | NA | SDM and uncertainty |
| Berntsen 2015 ^125^ | Review/content analysis | NA | General population | Variety | Patient goals |
| Blom et al 2016 ^126^ | RCT | Proactive care planning | Older people | Primary care | Other |
| Bookey-Bassett et al 2017 ^127^ | Concept analysis | NA | Older people | community | Interprofessional SDM |
| Bridges et al 2015 ^128^ | Qualitative | NA | HCPs | Secondary care - clinics | Influences on SDM, Other |

| **Author and year** | **If research give type** | **Description of intervention** | **Participants** | **Setting** | **Main focus** |
| --- | --- | --- | --- | --- | --- |
| Bugge 2006 ^129^ | Qualitative | NA | General population, HCPs | Community including outpatients | Barriers to SDM |
| Bynum 2014 ^130^ | Qualitative | NA | Older people | Community | Pt/carer views or preferences |
| Chong 2013b ^131^ | Qualitative | NA | HCPs | Primary & secondary care | Interprofessional SDM |
| Chong et al 2013 ^132^ | Qualitative | NA | HSCPs (multidisciplinary) | Primary & secondary care | Interprofessional SDM |
| Clayman 2016 ^177^ | Discussion | NA | NA | NA | Pt perspective of SDM |
| Col 2011 ^133^ | Discussion | NA | HCPs | Primary care | Interprofessional SDM |
| Cooper 2016 ^178^ | Discussion | NA | HCPs | Acute care | Goal concordance |
| Cramm 2012 ^135^ | Before/after | Chronic Care Model | HSCPs | Primary care | Service delivery for people with LTC |
| Cramm 2014 ^136^ | Mixed methods | Chronic Care Model | Older people | Primary care | Service delivery for people with LTC |
| Cramm 2016 ^134^ | Survey | Chronic care model | People with COPD (mostly older) | Community | Service delivery for people with LTC |
| **Author and year** | **If research give type** | **Description of intervention** | **Participants** | **Setting** | **Main focus** |
| Dardas, A. Z. et al 2016 ^137^ | Survey | NA | HCPs | Secondary care - orthopaedic clinic | Influences on SDM, Pt/carer views or preferences |
| Diabetes UK. 2011 ^20^ | Case studies | ‘Year of Care' model | HCPs, people with diabetes | Primary care | PCC in LTCs |
| Durand 2015 ^138^ | Documentary analysis | NA | Not applicable | Various | PDAs, incentives for SDM |
| Eaton 2015 ^176^ | Discussion | NA | Not applicable | Community | PCC in LTC |
| Edwards 2004 ^139^ | RCT | Training for GPs in SDM | GPs | Community, primary care | Education/training HCPs |
| Elwyn 2004 ^140^ | RCT | Training for GPs in SDM | General population, HCPs (GPs) | Community (primary care) | Education/training HCPs |
| Elwyn 2012 ^179^ | Discussion | NA | General population, HCPs | Any clinical consultation | Other, Relationships |
| Farrelly 2016 ^141^ | Qualitative | Joint care planning | People with mental health problems and HSCPs | Community | Care planning |
| Foot 2014 ^169^ | Review for guideline | NA | Not applicable | NA | Influences on SDM, Pt/carer views or preferences |
| **Author and year** | **If research give type** | **Description of intervention** | **Participants** | **Setting** | **Main focus** |
| Fried 2007 ^142^ | Observational | NA | Older people with COPD, cancer OR heart failure | Community | Influences on SDM |
| Gleason et al 2016 ^143^ | Survey | NA | Older people with multimorbidities | Community | PDAs |
| Glenpark Medical Practice 2016 ^26^ | Care study | The Year of Care initiative. | HCPs, People with LTC | Inner city | PCC for people with LTC |
| Gorin 2017 ^180^ | Discussion | NA | Not applicable | NA | Clinical nudges |
| Grim 2016 ^144^ | Qualitative | NA | People with mental health problems | Community | Influences on SDM |
| Groen-van de Ven 2016 ^145^ | Qualitative | NA | People with dementia, informal & paid carers. | Community | SDM for people with dementia |
| Hacking 2013 ^146^ | RCT | Pre-consultation coaching | Men with prostate cancer | Community | Coaching |
| Hart et al 2016 ^147^ | Qualitative | NA | Older people | Outpatient clinics | Use of PDA/tools |

| **Author and year** | **If research give type** | **Description of intervention** | **Participants** | **Setting** | **Main focus** |
| --- | --- | --- | --- | --- | --- |
| Health Foundation (also Baqir 2016) ^164,171^ | Case study | Multidisciplinary SDM | HSCPs, Resident and family members | Care home | Interprofessional SDM |
| Herlitz 2016 ^148^ | Qualitative | NA | Adolescents with diabetes | Diabetes clinic | Influences on SDM |
| Holmside Medical Group 2014 ^173^ | Case study | Holistic care for people with LTC | HCPs | Primary care | Service organisation |
| Jones 2011 ^149^ | Questionnaires | Tool for presenting risk | HCPs, people at risk of CVD | Clinics | Influences on SDM |
| Joseph-Williams 2017 ^150^ | Qualitative | SDM training | HCPs | Primary care | Training for HCPs, implementation of SDM |
| Körner 2013 ^151^ | Qualitative | NA | HCPs (multidisciplinary) | Primary & secondary care | Interprofessional SDM |
| Kuluski 2013 ^152^ | Qualitative | NA | Older people (2 or more LTC) | Uraban community in Ontario, Canada | Identifying pt goals |
| Ladin et al 2016 ^153^ | Qualitative | NA | Older people | Dialysis clinics | Interprofessional SDM |
| **Author and year** | **If research give type** | **Description of intervention** | **Participants** | **Setting** | **Main focus** |
| Légaré 2011a ^96^ | Qualitative | NA | HCPs (multidisciplinary) | Community | Influences on SDM, Relationships |
| Légaré 2011b ^181^ | Qualitative | NA | General population | Community | Interprofessional SDM |
| Lown 2011 ^172^ | Development of a model | NA | HCPs | Community or acute settings | Training for HCPs |
| Mercer 2016 ^154^ | Qualitative | Tool for providing PCC | Adults aged >30 years with multimorbidity. | Community | PCC |
| Naik et al 2016 ^182^ | Observational | NA | Cancer survivors with multimorbidities | Unsure | Pt/carer views or preferences |
| Nunes 2009 ^170^ | Guideline | NA | Not applicable | NA | Medicines adherence |
| Politi 2011 ^155^ | Development of a model | NA | Not applicable | NA | Collaborative decision making |
| Robben 2012 ^156^ | Qualitative | NA | Frail older people | Community | PDA |
| Ruggiano 2016 ^157^ | Qualitative | NA | Older people | Community | HCPs views on SDM |
| Sanders 2016 ^183^ | RCT | Training in SDM | GPs | Primary care | Training in SDM |
| Schaller 2015 & Schaller 2016 ^159,184^ | Before/after | e health portal | Family caregivers | Memory clinic in secondary care | Pt/carer views or preferences |
| **Author and year** | **If research give type** | **Description of intervention** | **Participants** | **Setting** | **Main focus** |
| Schuling et al 2012 ^163^ | Qualitative | NA | HCPs | Primary care | Patient engagement |
| Shay 2014 ^161^ | Qualitative | NA | General population | Community | Identifying pt goals |
| Sheaff 2017 ^162^ | Qualitative | NA | People aged >=65, with at least two LT health conditions | Primary care | Patient electronic records and PCC |
| Tietbohl 2015 ^167^ | Qualitative | Decision support intervention | HCPs | Community | PDA |
| van Summeren 2016 ^165^ | Questionnaire | Outcome Prioritisation Tool | Frailty, Multimorbidity | Primary care | Pt/carer views or preferences |
| Wrede-Sach 2013 ^166^ | Qualitative | NA | Older people | Community | Older people's experiences of SDM |
| Zoffman 2008 ^168^ | Qualitative | NA | PPl with DM and Nurses | Community & acute | Interprofessional SDM |
